# Does the pursuit of perfection by Chinese people harm interpersonal relationships? Evidence from the Wenjuan

**DOI:** 10.3389/fpsyg.2025.1505595

**Published:** 2025-04-02

**Authors:** Jun Zhang, Xiaoyan Luo, Wanshu Yang, Jie Xu, Ping Deng, Hui Wu, Junqiao Guo

**Affiliations:** ^1^Department of Education, Sehan University, Yeongam County, Republic of Korea; ^2^School of Business Administration, Tourism College of Zhejiang, Hangzhou, China; ^3^Department of Criminal Justice, Zhejiang Police Vocational Academy, Hangzhou, China; ^4^College of Tourism and Foreign Languages, Tourism College of Zhejiang, Hangzhou, China; ^5^School of Educational Science, Anhui Normal University, Wuhu, China

**Keywords:** perfectionism, peer relationships, psychological resilience, core self-evaluation, interpersonal sensitivity

## Abstract

**Objective:**

This study aims to explore the mechanisms of the relationship between Perfectionism and interpersonal relationships.

**Methods:**

Data were collected and research was conducted using the online platform Wenjuanxing, which distributed the Psychological Resilience Scale, Core Self-Evaluation Scale, Interpersonal Sensitivity Questionnaire, Perfectionism Scale, and Peer Relationships Scale.

**Results:**

Perfectionism not only directly and significantly predicts interpersonal sensitivity, but it can also mediate interpersonal sensitivity through dual or triple mediation paths formed by Psychological Resilience, Peer Relationships, and Core Self-Evaluation.

**Conclusion:**

Perfectionism can directly and significantly predict interpersonal sensitivity and can also indirectly predict interpersonal sensitivity through Psychological Resilience, Peer Relationships, and Core Self-Evaluation. The traditional Confucian educational philosophy in China advocates core values such as “benevolence,” “righteousness,” “propriety,” and “wisdom,” emphasizing that individuals should constantly cultivate themselves and their families to achieve moral perfection. This can lead to heightened interpersonal sensitivity and severe psychological repression. Modern education should build on traditional educational values while promoting inclusivity and tolerance, encouraging individuals to focus on the present, accept their emotions and experiences, respect human freedom of development, and safeguard psychological health.

## 1 Introduction

Perfectionism is a psychological trait in which individuals set high standards for themselves and others in the pursuit of perfection (Ma et al., 2010). It is often characterized by a strong focus on success and performance, along with a deep fear of failure (Conroy et al., 2007). Perfectionism can generally be divided into different dimensions, including self-oriented perfectionism (setting high standards for oneself), other-oriented perfectionism (setting high standards for others), and socially prescribed perfectionism (sensitivity to societal expectations) (Flett et al., 2016). Perfectionists typically have clear goal orientations, which allow them to focus better on tasks and increase work efficiency (Flett and Hewitt, 2002). However, when these goals are not achieved, perfectionists may severely criticize themselves, leading to low self-esteem and negative emotions (Luo et al., 2016). Some studies suggest that moderate perfectionism may motivate individuals to pursue excellence and enhance their sense of self-efficacy (Stoeber and Damian, 2014). On the other hand, high levels of perfectionism are often associated with psychological issues such as anxiety, depression, social anxiety, and interpersonal relationship difficulties (Flett et al., 2016).

Interpersonal sensitivity is a personality type characterized by excessive sensitivity to others’ behaviors and emotions (McCabe et al., 1999). This sensitivity allows individuals to more easily identify and interpret social cues and emotional states of others, but it may also lead them to overfocus on others’ perceptions and reactions in social situations (Hall et al., 2009). Prolonged attention to others’ emotions may result in emotional exhaustion for the individual, negatively impacting their mental health (Zhang, 2024). Interpersonal sensitivity is often closely linked to emotional intelligence, empathy, and self-awareness. Highly sensitive individuals are better at understanding others’ emotions and are skilled in using interpersonal communication techniques such as verbal communication, body language, active listening, and feedback, leading to higher-quality interpersonal interactions (Erozkana, 2013; Acevedo et al., 2014). These individuals are more adept at recognizing potential conflicts and addressing them early, thus preventing possible interpersonal disputes (Trnka, 2013). However, this heightened sensitivity can also lead to anxiety and discomfort in social situations, as these individuals may worry about being misunderstood or judged (Wilhelm et al., 2004).

### 1.1 The relationship between perfectionism and interpersonal sensitivity

Self-determination theory emphasizes the importance of self-assessment and external evaluation for an individual’s mental health. Perfectionists often rely on external feedback to confirm their self-worth, which makes them exceptionally sensitive to others’ opinions, increasing the vulnerability of their interpersonal relationships (Vicent et al., 2020). For example, when individuals feel that they have autonomy in setting goals (Sheldon, 2014), especially when they perceive support for their autonomy from significant people in their lives, their goal pursuit tends to be successful. On the other hand, if they do not receive support from others, they may feel more discouraged and withdraw from social interactions (Moore et al., 2018). A study using the Self-Rating Symptoms Scale (SCL-90) and the Chinese version of the Frost Multidimensional Perfectionism Scale, revised by Yang Hongfei, surveyed 325 medical students. The results indicated a significant correlation between perfectionism and interpersonal sensitivity, which is consistent with findings from other researchers (Yang and Zhang, 2004; Xie, 2004). Moreover, some studies have shown that perfectionism is closely related to peer relationships (Huang, 2024), psychological resilience (Wang, 2019), and core self-evaluation (Zhang et al., 2016). Based on this, the present study proposes Hypothesis 1: Perfectionism can significantly predict interpersonal sensitivity.

### 1.2 The indirect effect of peer relationships

Peer relationships refer to the relationships between individuals of the same age or with similar levels of psychological development. These are parallel, equal relationships (Xiao et al., 2024). Such relationships are typically built on shared interests, values, and experiences, carrying significant social and emotional meaning. Peer relationships generally include three aspects: emotional support, social interaction, and mutual assistance and cooperation. Emotional support refers to the care and support provided by peers to help each other cope with life’s challenges. Social interaction involves frequent exchanges between peers, including communication, activities, and collaboration, which strengthen the sense of closeness in the relationship. Mutual assistance and cooperation refer to the collaboration and support between peers in learning, work, or leisure activities, which fosters mutual development (Reiter and Weller, 2020).

Good peer relationships help individuals develop social skills and communication abilities, reduce the risk of anxiety and depression, enhance overall mental health, and boost self-esteem and self-efficacy (Yan, 2019). In contrast, poor peer relationships may lead to conflicts and competition, which can result in emotional stress and psychological burden (Xiang et al., 2022). Studies have shown that perfectionists often experience higher anxiety and lower satisfaction in peer relationships due to their excessive expectations of others and themselves, which can lead to social avoidance and interpersonal conflicts (Flett and Hewitt, 2002). The quality of peer relationships is closely related to an individual’s interpersonal sensitivity (Liu et al., 2019). Based on this, the present study proposes Hypothesis 2: Peer relationships mediate the relationship between perfectionism and interpersonal sensitivity.

### 1.3 The indirect effect of core self-evaluation

There is a close relationship between perfectionism and core self-evaluation. Perfectionists typically have a higher sense of self-efficacy (Flett and Hewitt, 2002; Sun, 2024), but they are more likely to experience frustration when faced with failure, which can negatively affect their core self-evaluation (Yu, 2019).

Core self-evaluation refers to an individual’s fundamental assessment of their abilities and worth (Mubarak and Quinn, 2019). It reflects how individuals perceive themselves and how this self-perception impacts their behavior and mental health. Core self-evaluation primarily includes four dimensions: self-esteem, self-efficacy, emotional stability, and internal locus of control. According to social comparison theory, perfectionists tend to engage in upward comparisons with others, leading to a more negative core self-evaluation. This comparison not only affects self-esteem but can also lead to anxiety and depression (Festinger, 1954). Higher core self-evaluation is associated with better mental health, helping to reduce anxiety and depression (Wu et al., 2024). It also promotes greater confidence and initiative in the workplace, enhancing work efficiency (Liu, 2024), and contributes to the development of positive interpersonal relationships and social interactions (Lei et al., 2018). However, blind overconfidence may lead to arrogance (Dunning et al., 2003; Wen et al., 2016), which can reduce sensitivity to feedback from others (Li, 2022). Based on this, the present study proposes Hypothesis 3: Core self-evaluation mediates the relationship between perfectionism and interpersonal sensitivity.

### 1.4 The indirect effect of psychological resilience

Psychological resilience refers to an individual’s ability to adapt, recover, and maintain mental health in the face of adversity, stress, or challenges (Ma et al., 2024). This ability enables individuals to effectively cope with difficulties in life and return to a normal state after experiencing trauma. Psychological resilience typically includes four dimensions: emotional regulation, self-efficacy, adaptability, and social support. Psychological resilience helps reduce the risk of anxiety and depression and promotes overall mental health (Anna, 2024).

Perfectionists tend to have higher self-efficacy, which may enhance their psychological resilience (Stoeber and Damian, 2014). Attachment theory suggests that individuals with high psychological resilience often possess a secure attachment style, which makes them more resilient when facing challenges in interpersonal relationships. In contrast, individuals with high interpersonal sensitivity, due to insecure attachment styles, may over-interpret others’ responses, leading to emotional instability (Mikulincer and Shaver, 2007). Individuals with high psychological resilience are more likely to maintain positive coping strategies under stress (Aldao et al., 2010), which helps them maintain a positive attitude in social situations, strengthening their connections and support from others. Research has shown that high psychological resilience is typically associated with lower interpersonal sensitivity, meaning that individuals with high psychological resilience are more adaptable when facing others’ evaluations. On the other hand, individuals with low psychological resilience may be overly sensitive to others’ opinions, leading to social anxiety and interpersonal problems (Liang et al., 2019). Based on this, the present study proposes Hypothesis 4: Psychological resilience mediates the relationship between perfectionism and interpersonal sensitivity.

### 1.5 The potential indirect effect of peer relationships and psychological resilience

Social support theory suggests that various types of social support, including peer relationships, not only alleviate psychological stress but also enhance an individual’s positive coping abilities, promoting the development of psychological resilience (Sippel et al., 2015). Perfectionists’ pursuit of higher-quality friendships may make them overly critical in their interactions with others, which can hinder the healthy development of peer relationships (Flett et al., 2016), potentially weakening an individual’s psychological resilience and reducing their adaptability in the face of difficulties (Leng, 2020). Research has found a significant negative correlation between psychological resilience and interpersonal sensitivity, with individuals possessing high psychological resilience generally being less sensitive to others’ opinions (Zhang, 2015). Based on this, the present study proposes Hypothesis 5: Peer relationships and psychological resilience mediate the relationship between perfectionism and interpersonal sensitivity.

### 1.6 The potential indirect effect of peer relationships and core self-evaluation

Perfectionists may face more challenges in peer relationships. Their perfectionistic tendencies may lead them to have excessively high expectations of others, which can affect the quality and satisfaction of friendships (Hewitt and Flett, 1991), often resulting in feelings of isolation and dissatisfaction in their relationships with peers. For example, perfectionists may experience higher anxiety in social interactions, increasing the likelihood of conflicts and misunderstandings (Flett et al., 2016). Cognitive behavioral theory suggests that attention should be given to how an individual’s thought patterns influence their emotions and behaviors. In peer relationships, positive interactions and feedback can enhance an individual’s self-efficacy, thereby improving their core self-evaluation (Ren, 2017). In contrast, negative social experiences may lead to negative self-evaluations (Beck, 1976; Brockner et al., 2004). Improving core self-evaluation is crucial for an individual, as it may help reduce interpersonal sensitivity and improve the quality of social interactions (Judge et al., 2003).Based on this, the present study proposes Hypothesis 6: Peer relationships and core self-evaluation mediate the relationship between perfectionism and interpersonal sensitivity.

### 1.7 The potential indirect effect of psychological resilience and core self-evaluation

Research has shown that individuals with high levels of perfectionism often exhibit strong psychological resilience because they set high goals and actively confront challenges (Stoeber and Damian, 2014). Resilience theory explores the adaptability individuals demonstrate in the face of adversity. Individuals with strong psychological resilience are typically able to cope positively with stress and challenges, and this ability is closely linked to their core self-evaluation (Masten, 2001). Individuals with higher psychological resilience tend to have higher core self-evaluation, which enables them to show greater adaptability in adversity (Chen, 2014) and to engage confidently in social interactions without worrying about deteriorating relationships (Schmader et al., 2008). Based on this, the present study proposes Hypothesis 7: Psychological resilience and core self-evaluation mediate the relationship between perfectionism and interpersonal sensitivity.

### 1.8 The potential indirect effects of peer relationships, psychological resilience, and core self-evaluation

Research has shown that perfectionistic tendencies can influence an individual’s performance in social interactions. Perfectionists often have excessively high expectations of themselves and others, which leads to more challenges when establishing and maintaining peer relationships (Hewitt and Flett, 1991). Negative peer relationships make it difficult to gain emotional support (Katz and Arieli, 2013), which can lower an individual’s psychological resilience (Windle, 2011). As a result, individuals may feel less confident and have lower self-evaluations.

Some studies have found a significant negative correlation between core self-evaluation and interpersonal sensitivity. Individuals with high core self-evaluation tend to be less sensitive to others’ feedback and are more confident in handling various social situations, while individuals with low core self-evaluation may be overly sensitive to others’ opinions, leading to social anxiety (Schmader et al., 2008). Based on this, the present study proposes Hypothesis 8: Peer relationships, psychological resilience, and core self-evaluation mediate the relationship between perfectionism and interpersonal sensitivity.

## 2 Methods

### 2.1 Participants

Wenjuanxing, an online data collection, analysis, and management platform founded by Hu Xiao and Wu Yong in November 2006, is the most widely used social survey data platform in China. Since its official operation 18 years ago, the platform has released 284 million questionnaires and collected 21.922 billion responses. From November 10, 2023, to February 10, 2024, this study conducted an online survey through the Wenjuanxing platform, obtaining 556 valid responses. During the three-month online survey, the ratio of male to female participants was 36.51 to 63.49%, with 21.77% of participants from urban areas and 78.23% from rural areas.

### 2.2 Research tools

#### 2.2.1 Psychological Resilience Scale

The Psychological Resilience Scale developed by Hu & Gan was used in this study. The scale consists of two dimensions with a total of 27 items. It uses a 5-point Likert scale, where each item has five response options for participants to choose from: Strongly disagree (1 point), Disagree (2 points), Uncertain (3 points), Agree (4 points), and Strongly agree (5 points). Items 1, 2, 5, 6, 9, 12, 15, 16, 17, 21, 26, and 27 are scored reversely, while the remaining items are scored positively. The sum of the scores for all items represents the total score, with a higher total score indicating better psychological resilience (Hu and Gan, 2008). In this study, reliability and validity tests were conducted, and the Cronbach’s alpha for this scale was 0.90. The structural validity indicators were good: CFI = 0.91, TLI = 0.90, RMSEA = 0.07, SRMR = 0.06.

#### 2.2.2 Core Self-Evaluation Scale

The Core Self-Evaluation Scale, translated and revised by Du, Zhang, and Zhao, was used in this study. The revised scale consists of two dimensions with a total of 10 items, using a 5-point Likert scale ranging from “Strongly disagree” to “Strongly agree,” with scores of 1–5. There are 5 positively scored items and 5 reversely scored items. The higher the participant’s score, the higher their core self-evaluation (Du et al., 2012). In this study, reliability and validity tests were conducted, with the Cronbach’s alpha for the scale being 0.83. The structural validity indicators were good: CFI = 0.98, TLI = 0.97, RMSEA = 0.06, SRMR = 0.02.

#### 2.2.3 Interpersonal Sensitivity Questionnaire

We used the interpersonal sensitivity dimension from the SC1-90 Symptom Self-Rating Scale to measure interpersonal sensitivity, which includes 9 items. The scale uses a 5-point rating system, where participants select one option from the five provided: “None” (1 point), “Mild” (2 points), “Moderate” (3 points), “Quite severe” (4 points), and “Severe” (5 points). In this study, we used the total score of interpersonal sensitivity as the rating criterion, where higher scores indicate more prominent interpersonal sensitivity issues (Wang et al., 1999). In this study, we conducted reliability and validity tests, with the Cronbach’s alpha for the scale being 0.95. The structural validity indicators were good: CFI = 0.98, TLI = 0.97, RMSEA = 0.07, SRMR = 0.02.

#### 2.2.4 Perfectionism Scale

We used the revised Chinese version of the Frost Multidimensional Perfectionism Scale (Zi and Zhou, 2006) as the measurement tool. This scale was revised based on a sample of undergraduate students from mainland China and uses a 5-point rating system, ranging from “Strongly disagree” to “Strongly agree,” with scores from 1 to 5. The scale consists of 26 items, all scored positively. The total score is the sum of the individual item scores, with higher scores indicating higher levels of perfectionism (Zi and Zhou, 2006). In this study, we conducted reliability and validity tests, with the Cronbach’s alpha for the scale being 0.93. The structural validity indicators were good: CFI = 0.91, TLI = 0.90, RMSEA = 0.07, SRMR = 0.04.

#### 2.2.5 Peer Relationships Scale

The Peer Relationships Scale used in this study is a revised version of the Peer Relationships dimension from the Interpersonal Sensitivity Scale developed by Wo et al. (2001). It includes two dimensions with a total of 17 items. The scale uses a Likert 5-point rating system, where each item has 5 options: “Strongly disagree” (1 point), “Disagree” (2 points), “Neutral” (3 points), “Agree” (4 points), and “Strongly agree” (5 points). A higher score indicates poorer peer relationships, while a lower score indicates better peer relationships (Wo et al., 2001). In this study, the reliability of the scale was Cronbach’s alpha = 0.95, and the structural validity indicators were good: CFI = 0.95, TLI = 0.94, RMSEA = 0.08, SRMR = 0.03.

#### 2.2.6 Data analysis strategy

We used SPSS 25.0 to calculate the means, standard deviations, reliability coefficients, and correlation coefficients for Perfectionism, Psychological Resilience, Interpersonal Sensitivity, Core Self-Evaluation, and Peer Relationships. This helped us understand the levels of these variables among Chinese individuals. Confirmatory factor analysis (CFA) of the scales was conducted using Mplus 8.0 to test the internal structural validity of the scales. In evaluating model fit indices, we required the following criteria for the model’s fit: RMSEA <0.1, SRMR <0.1, TLI > 0.9, CFI > 0.9 (Brown, 2015).

In exploring the relationships between variables, we used Mplus 8.0 to construct structural equation models, which were analyzed in two main procedures. We established a structural equation model with Perfectionism as the independent variable, Interpersonal Sensitivity as the dependent variable, and Peer Relationships, Psychological Resilience, and Core Self-Evaluation as mediating variables. For testing the significance of both independent indirect effects and chain indirect effects, we used the Bootstrap method with a 95% confidence interval for bias-corrected bootstrapping. Indirect effects were considered significant if the confidence interval did not include zero.

## 3 Results

### 3.1 Common method bias test

when researchers use a single data collection method (such as a questionnaire), respondents may answer multiple scales at the same time, and the way questions are phrased and ordered can lead to higher consistency in responses (Yu, 2012). To avoid common method bias in this study, we used Harman’s single-factor method for exploratory factor analysis (Zhang et al., 2024). A total of 16 factors with eigenvalues greater than 1 were obtained, with the first factor explaining 26.02% of the total variance, which is below the 40% threshold (Guo et al., 2023), indicating that there is no significant common method bias.

### 3.2 Descriptive statistics and correlation analysis

We calculated the mean, standard deviation, correlation coefficient, skewness, and kurtosis for the scores of Perfectionism, Psychological resilience, interpersonal sensitivity, Core self-evaluation, and Peer relationships. In the normality test of the data in this study, we used the criterion that the absolute value of skewness should be less than 2 or the absolute value of kurtosis should be less than 7 to determine if the data follows a normal distribution (Kim, 2013). The results showed that the absolute values of the skewness and kurtosis for the five variables in this study were within the acceptable range, indicating that the collected data approximates a normal distribution and is suitable for statistical analysis.

Gender was significantly positively correlated with Peer relationships (*r* = 0.11, *p* < 0.05) and negatively correlated with Core self-evaluation (*r* = −0.09, *p* < 0.05), but showed no significant correlation with Perfectionism, interpersonal sensitivity, and Psychological resilience (*p* > 0.05). Household registration was significantly positively correlated with Psychological resilience (*r* = 0.10, *p* < 0.05), but showed no significant correlation with Peer relationships, Core self-evaluation, Perfectionism, and interpersonal sensitivity (*p* > 0.05). Peer relationships were significantly positively correlated with Perfectionism (*r* = 0.31, *p* < 0.05) and interpersonal sensitivity (*r* = 0.56, *p* < 0.05), and significantly negatively correlated with Psychological resilience (*r* = −0.45, *p* < 0.05) and Core self-evaluation (*r* = −0.59, *p* < 0.05). Perfectionism showed no significant correlation with Psychological resilience (*r* = −0.03, *p* > 0.05), but was significantly positively correlated with interpersonal sensitivity (*r* = 0.33, *p* < 0.05) and negatively correlated with Core self-evaluation (*r* = −0.18, *p* < 0.05). Psychological resilience was significantly positively correlated with Core self-evaluation (*r* = 0.68, *p* < 0.05) and negatively correlated with interpersonal sensitivity (*r* = −0.41, *p* < 0.05). There was a significant negative correlation between interpersonal sensitivity and Core self-evaluation (*r* = −0.54, *p* < 0.05), as shown in Table 1.

**Table 1:** Means, standard deviations, correlation coefficients, skewness, and kurtosis of variables.

	1	2	3	4	5	6	7
1. Gender	1						
2. Household registration	0.00	1					
3. Peer relationships	0.11**	0.02	1				
4. Perfectionism	0.03	0.00	0.31**	1			
5. Psychological resilience	−0.04	0.10*	−0.45**	−0.03	1		
6. Interpersonal sensitivity	0.01	0.01	0.56**	0.33^**^	−0.41^**^	1	
7. Core self-evaluation	−0.09*	0.07	−0.59**	−0.18^**^	0.68^**^	−0.54^**^	1
Quantity			556	556	556	556	556
Minimum value			17.00	27.00	68.00	9.00	10.00
Maximum value			60.00	134.00	130.00	45.00	50.00
Mean			34.59	74.05	87.08	16.91	34.84
Standard deviation			13.02	15.52	13.38	6.78	6.49
Skewness			0.01	−1.16	1.12	0.66	0.59
Standard error of skewness			0.10	0.10	0.10	0.10	0.10
Kurtosis			−1.52	1.92	0.65	−0.01	0.15
Standard error of kurtosis			0.20	0.20	0.20	0.20	0.20

*N* = 556, *p < 0.05, **p < 0.01, ***p < 0.001.

We used independent sample t-tests to examine the differences in Perfectionism, Psychological resilience, interpersonal sensitivity, Core self-evaluation, and Peer relationships between individuals with different household registrations and genders. Regarding gender, there were significant differences between male and female participants in Peer relationships (*t* = −2.75, *p* < 0.05) and Core self-evaluation (*t* = 2.25, *p* < 0.05), but no significant differences in Perfectionism (*t* = −0.79, *p* > 0.05), Psychological resilience (*t* = 1.07, *p* > 0.05), and interpersonal sensitivity (*t* = −0.43, *p* > 0.05). Regarding household registration, there was a significant difference between rural and urban residents in Psychological resilience (*t* = −2.15, *p* < 0.05), but no significant differences in Perfectionism (*t* = −0.15, *p* > 0.05), interpersonal sensitivity (*t* = −0.43, *p* > 0.05), Core self-evaluation (*t* = −1.52, *p* > 0.05), and Peer relationships (*t* = −0.60, *p* > 0.05), as shown in Table 2.

**Table 2:** Differences in perfectionism, psychological resilience, interpersonal sensitivity, core self-evaluation, and peer relationships among subjects by gender and household registration.

Dependent variable	Independent variable		*F*	Significance	*t*	Sig (two-tailed)
Peer relationships	Gender	Assume equal variance	0.07	0.78	−2.75	0.00
		Do not assume equal variance			−2.74	0.00
Perfectionism		Assume equal variance	10.25	0.00	−0.83	0.40
		Do not assume equal variance			−0.79	0.42
Psychological resilience		Assume equal variance	3.33	0.06	1.07	0.28
		Do not assume equal variance			1.05	0.29
Interpersonal sensitivity		Assume equal variance	8.84	0.00	−0.43	0.66
		Do not assume equal variance			−0.42	0.67
Core self-evaluation		Assume equal variance	3.21	0.07	2.25	0.02
		Do not assume equal variance			2.20	0.02
Peer relationships	Household registration	Assume equal variance	0.47	0.49	−0.60	0.54
		Do not assume equal variance			−0.59	0.55
Perfectionism		Assume equal variance	0.02	0.88	−0.15	0.88
		Do not assume equal variance			−0.15	0.88
Psychological resilience		Assume equal variance	16.48	0.00	−2.47	0.01
		Do not assume equal variance			−2.15	0.03
Interpersonal sensitivity		Assume equal variance	0.45	0.50	−0.43	0.66
		Do not assume equal variance			−0.43	0.66
Core self-evaluation		Assume equal variance	6.44	0.01	−1.65	0.09
		Do not assume equal variance			−1.52	0.12

We used one-way analysis of variance (ANOVA) to examine the differences in Perfectionism, Psychological resilience, interpersonal sensitivity, Core self-evaluation, and Peer relationships across different age groups. The results showed that there were no significant differences between different age groups in Perfectionism (*F* = 1.063, *p* > 0.05), Psychological resilience (*F* = 0.668, *p* > 0.05), interpersonal sensitivity (*F* = 1.863, *p* > 0.05), Core self-evaluation (*F* = 0.141, *p* > 0.05), and Peer relationships (*F* = 0.155, *p* > 0.05), as shown in Table 3.

**Table 3:** Age differences in perfectionism, psychological resilience, interpersonal sensitivity, core self-evaluation, and peer relationships among participants.

		Sum of squares	Degrees of freedom	Mean square	*F*	Significance
Peer relationships	Between-group	52.77	2	26.38	0.15	0.85
	Within-group	94056.97	553	170.08		
Perfectionism	Between-group	512.14	2	256.07	1.06	0.34
	Within-group	133171.34	553	240.81		
Psychological resilience	Between-group	243.13	2	121.56	0.66	0.51
	Within-group	100567.35	553	181.85		
Interpersonal sensitivity	Between-group	171.05	2	85.52	1.86	0.15
	Within-group	25390.30	553	45.91		
Core self-evaluation	Between-group	11.88	2	5.94	0.14	0.86
	Within-group	23385.42	553	42.28		

### 3.3 Construction and testing of the structural equation model

**Figure 1:**
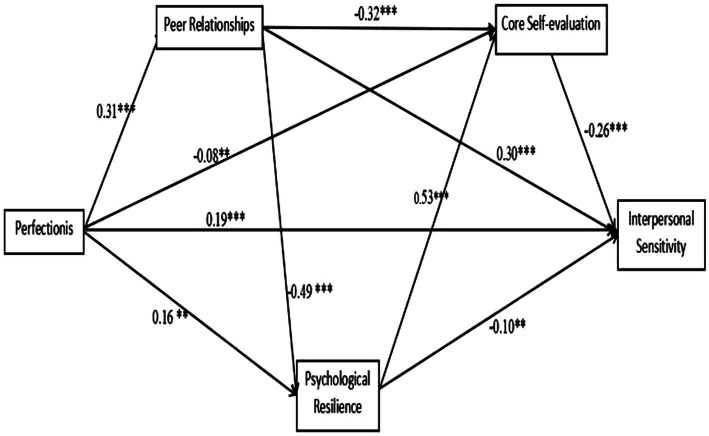
The mediating effect model of peer relationships, core self-evaluation, and psychological resilience in the relationship between perfectionism and interpersonal sensitivity.

We constructed a model in which Perfectionism is the independent variable, interpersonal sensitivity is the dependent variable, and Peer relationships, Core self-evaluation, and Psychological resilience are the mediating variables. The results of the confirmatory factor analysis showed that the model fit indices were good: RMSEA = 0.06, SRMR = 0.03, TLI = 0.96, CFI = 0.98. Perfectionism had a significant direct effect on interpersonal sensitivity (*β* = 0.19, *p* < 0.001). Perfectionism positively predicted Peer relationships (*β* = 0.31, *p* < 0.001), Psychological resilience (*β* = 0.16, *p* < 0.01), and Core self-evaluation (*β* = −0.08, *p* < 0.01). Peer relationships negatively predicted Core self-evaluation (*β* = −0.32, *p* < 0.001) and Psychological resilience (*β* = −0.49, *p* < 0.001), and positively predicted interpersonal sensitivity (*β* = 0.30, *p* < 0.001). Psychological resilience positively predicted Core self-evaluation (*β* = 0.53, *p* < 0.001), and Core self-evaluation negatively predicted interpersonal sensitivity (*β* = −0.26, *p* < 0.001), as shown in Figure 1. Bootstrap sampling with 1,000 resamples was used to test the significance of the indirect effects. The results showed that the 7 mediating paths we constructed had 95% confidence intervals that did not contain 0, indicating that all 7 mediating paths reached a significant level, as shown in Table 4.

**Table 4:** Bootstrap analysis for significance testing of mediation effects.

Mediation pathway	Effect size	95% Confidence interval	
		Lower bound	Upper bound
Perfectionism-Peer relationships-interpersonal sensitivity	0.04	0.02	0.06
Perfectionism-Core self-evaluation-interpersonal sensitivity	0.01	0.00	0.02
Perfectionism-Psychological resilience-interpersonal sensitivity	0.00	−0.01	0.00
Perfectionism-Peer relationships-Psychological resilience-interpersonal sensitivity	0.00	0.00	0.01
Perfectionism-Peer relationships-Core self-evaluation-interpersonal sensitivity	0.01	0.00	0.01
Perfectionism-Psychological resilience-Core self-evaluation-interpersonal sensitivity	−0.01	−0.02	0.00
Perfectionism-Peer relationships-Psychological resilience-Core self-evaluation-interpersonal sensitivity	0.01	0.00	0.02

## 4 Discussion

This study found that Perfectionism can significantly predict interpersonal sensitivity, supporting Hypothesis 1 and aligning with self-determination theory. The cognitive model suggests that it is not the situation itself that affects people’s feelings, but rather how they interpret the situation. Individuals with perfectionistic tendencies may experience anxiety and self-doubt due to setting high standards. They tend to engage in harsh self-criticism, and their sense of self-worth is often dependent on external validation and feedback from others (Sharon et al., 2014). This can lead to an exaggerated sensitivity to their own or others’ reactions (Fenn and Byrne, 2013). This dependency makes them highly sensitive to others’ perceptions, sometimes viewing others’ evaluations as direct reflections of their own value. When they perceive negative feedback from others, they may experience strong emotional reactions (Liang, 2023). Perfectionists often set unrealistic standards and expectations, not only for themselves but also for others. They hold similarly stringent expectations regarding others’ behaviors and reactions. When others’ performances fail to meet these expectations, they are likely to feel disappointed and dissatisfied in social interactions (Stoeber, 2014). Prolonged stress in perfectionists can significantly affect their physical health (Soo et al., 2009; Johnson et al., 2014). This study suggests that each person is an independent individual, with their thoughts and beliefs influenced by their social environment, family background, and education. As a result, individuals’ ways of thinking inevitably differ to some degree. Striving to improve oneself is a positive behavior that benefits personal growth and development. However, excessive pursuit of perfection can not only be detrimental to one’s mental and physical health but can also harm relationships with others. The correct mindset should be that if effort does not lead to satisfactory results, one should learn to be forgiving of oneself. Similarly, we should be tolerant of those around us and accept that they may not be the same as us.

This study found that Perfectionism can significantly predict interpersonal sensitivity through independent indirect effects via Peer relationships, Core self-evaluation, and Psychological resilience, supporting Hypotheses 2, 3, and 4, as well as supporting social comparison theory and attachment theory. Firstly, Perfectionism significantly predicts interpersonal sensitivity through Peer relationships. The multidimensional Perfectionism model proposed by Hewitt and Flett suggests that Perfectionism not only impacts individuals’ self-assessment but also plays a significant role in interpersonal relationships. Highly perfectionistic individuals often exhibit competitive behaviors to maintain their self-image, which can cause discomfort among peers. In order to maintain their Peer relationships, they become more focused on others’ opinions and reactions (Hewitt et al., 2002). They tend to impose high expectations on others, which may provoke others’ resentment, thereby making interpersonal relationships more tense and sensitive (Kang and Chen, 2012). However, when they notice their peers outperforming them, they may engage in self-deprecation, causing a decrease in their self-confidence and social abilities (Flett et al., 2016). It should be noted, however, that in this study, the researchers found a positive correlation between Perfectionism and Peer relationships. This may be due to the sample size. Future research should employ larger sample sizes through surveys and interviews, which may yield more scientifically accurate results. Additionally, the participants in this study may not be extreme perfectionists; their competitive behavior may not yet severely damage their Peer relationships, which might explain the observed positive correlation. Secondly, Perfectionism significantly predicts interpersonal sensitivity through Core self-evaluation. The Core self-evaluation model suggests that while perfectionists may have intrinsic achievement motivation that helps maintain a positive mindset, the high standards and expectations they set for themselves are often difficult to achieve. This can lead to excessive self-doubt, affecting their Core self-evaluation (Hewitt and Flett, 1991). As a result, they may exhibit higher levels of self-criticism (Flett and Hewitt, 2002; Wang, 2023). Generally, when individuals hold negative views about their own abilities and worth, they may feel anxious and insecure in social situations. This anxiety makes them more sensitive to others’ reactions and increases their concern about interpersonal relationships (Schunk and Zimmerman, 2008). Thirdly, Perfectionism significantly predicts interpersonal sensitivity through Psychological resilience. The motivation and adaptation model emphasizes how an individual’s motivation affects their adaptability, especially when facing challenges and stress. For perfectionists, intrinsic achievement motivation drives them to pursue high standards, while maintaining a positive mindset in the face of failure, thus enhancing their resilience (Egan et al., 2011). In other words, this motivation is often associated with positive self-improvement and Psychological resilience (Zhou, 2014). The stronger the individual’s achievement motivation, the higher their Psychological resilience tends to be, making them more capable in coping strategies, stress management, and negative emotion regulation. This enables them to demonstrate greater confidence and stability in social interactions, reducing excessive sensitivity to interpersonal relationships (Chen, 2014). In fact, Chinese society has always placed great importance on interpersonal communication, yet surprisingly, the education system in China has not emphasized the cultivation of this quality in children. With the rapid development of the information age, more and more Chinese youth have become addicted to online games, reducing their opportunities for social interaction, which increases difficulties in their interpersonal relationships (Li, 2016). The number of individuals experiencing psychological issues has also risen sharply, leading to confusion and a lack of solutions in current mental health institutions (Li and Du, 2024). We believe that families, schools, and society should take on this responsibility, strictly limit online game services for minors, and encourage participation in social activities. This would be greatly beneficial for improving social skills.

This study found that the “Peer relationships-Psychological resilience,” “Psychological resilience-Core self-evaluation,” and “Psychological resilience-Core self-evaluation” dual indirect effects significantly predicted interpersonal sensitivity, supporting Hypotheses 5, 6, and 7, as well as supporting social support theory, resilience theory, and cognitive-behavioral theory. Firstly, the “Peer relationships-Psychological resilience” dual indirect effect plays a significant role in the relationship between Perfectionism and interpersonal sensitivity. Research shows that perfectionists, due to their tendency to hide their vulnerabilities and problems, may reduce opportunities to receive emotional support from peers (Stoeber et al., 2015). This leads them to feel more isolated in the face of stress and lack support from those around them. Emotional support from peers not only significantly enhances their Psychological resilience and reduces anxiety but also improves their coping ability in social interactions (Allen et al., 2016). Secondly, the “Psychological resilience-Core self-evaluation” dual indirect effect plays a significant role in the relationship between Perfectionism and interpersonal sensitivity. Chen et al. (2018) pointed out that perfectionists may struggle with emotional regulation, leading them to exhibit lower Psychological resilience under stress, which in turn affects their mental health (Chen et al., 2018). Individuals with low Psychological resilience tend to show negative self-perception when facing challenges, and this cognitive feedback contributes to lowering their Core self-evaluation (Tugade and Fredrickson, 2004). As a result, individuals often lack confidence in interpersonal communication, struggle with complex social relationships, and are more sensitive to interpersonal issues (Luthans et al., 2006). In the three significant dual-mediation paths between Perfectionism and interpersonal sensitivity, we must emphasize the roles played by Peer relationships, Psychological resilience, and Core self-evaluation, especially since there is also significant influence between these variables. This study suggests that Confucianism has influenced Asian countries for over 2,000 years, and there is a widespread pursuit of perfection in Chinese thought, which brings immense psychological pressure. However, the negative impact felt by different individuals varies, and this is mainly due to differences in each person’s social support system and psychological resilience (Deng, 2017). It is recommended that the Chinese education system should place more emphasis on teaching children emotional management skills and techniques, such as mindfulness, meditation, and relaxation techniques, as these measures can effectively help them maintain psychological balance.

This study found that the “Peer relationships-Psychological resilience-Core self-evaluation” triple indirect effect significantly predicted interpersonal sensitivity, supporting Hypothesis 8. Flett and Hewitt (2002) pointed out that perfectionists tend to feel uneasy and anxious in social situations, which may lead them to avoid social interactions, thus limiting their contact and communication with peers (Flett and Hewitt, 2002). This may result in individuals experiencing higher emotional stress, lowering their Psychological resilience and coping abilities (Hawkins et al., 2015), and making it more likely for them to lose control over negative emotions, which in turn leads to greater interpersonal sensitivity (Luthans et al., 2006).

## 5 Limitations and implications

We must acknowledge several limitations in this study. First, the sample size collected through an online format is relatively small, and the sample may not be fully representative. Second, the data used in this study are cross-sectional, which may not accurately reflect the causal relationships between variables. Therefore, future research could explore cross-lagged studies to investigate the relationships between variables over time.

## 6 Conclusion

Perfectionism in Chinese individuals not only directly predicts interpersonal sensitivity but also predicts it through the indirect effects of peer relationships, psychological resilience, and core self-evaluation. Confucius, a prominent figure in Chinese Confucianism, advocated for individuals to become morally virtuous and emphasized the importance of etiquette in interpersonal interactions. While the pursuit of high standards is admirable, excessive perfectionism and the pressure to maintain relationships can make interpersonal connections in China more complex and hinder psychological growth in highly stressful situations. From this perspective, Chinese society should promote moderate self-requirements and a tolerant attitude toward life and interpersonal relationships in order to address the increasingly prominent mental health issues in recent years.

## Data Availability

The raw data supporting the conclusions of this article will be made available by the authors, without undue reservation.
